# Novel growth pattern‐specific digital marker of TILs improves stratification of lung adenocarcinoma patients

**DOI:** 10.1002/path.6498

**Published:** 2025-11-15

**Authors:** Arwa AlRubaian, Ayesha Azam, Nasir M Rajpoot, Shan E Ahmed Raza

**Affiliations:** ^1^ Tissue Image Analytics Centre, Department of Computer Science University of Warwick Coventry UK; ^2^ Department of Computer Science King Saud University Riyadh Saudi Arabia; ^3^ Department of Cellular Pathology University Hospitals Coventry and Warwickshire NHS Trust Coventry UK; ^4^ Histofy Ltd Coventry UK

**Keywords:** lung adenocarcinoma, tumour microenvironment, histological growth patterns, survival analysis, artificial intelligence, TILs, STILs, lymphocytes, histology images

## Abstract

Lung adenocarcinoma (LUAD) is one of the most prevalent forms of cancer and continues to be associated with high mortality rates, despite recent advances in cancer therapy. Effective risk stratification is critical for guiding treatment decisions and improving our understanding of disease mechanisms. However, current prognostic approaches face considerable limitations. Growth pattern‐based grading serves as a prognostic indicator of tumour aggressiveness, but is inherently subjective and prone to a high degree of variability among observers. Other well‐established prognostic indicators, such as tumour infiltrating lymphocytes (TILs) and stromal TILs (sTILs) scores, provide valuable prognostic information but require labour‐intensive assessment. The pronounced heterogeneity of LUAD further complicates prognosis and underscores the need for robust, integrative biomarkers that capture both the morphological and immunological characteristics of the tumour. To address this need, we propose an AI‐based growth‐pattern‐specific TILs (GPS‐TILs) marker that quantifies TILs and sTILs within each growth pattern separately. By integrating morphological information from the tumour growth patterns and immune microenvironment data from TILs, we demonstrate that the proposed GPS‐TILs marker improves patient stratification. We evaluated the prognostic utility of GPS‐TILs using survival analysis with Cox proportional hazards models in a cross‐validation setting using The Cancer Genome Atlas LUAD (TCGA‐LUAD) cohort. Our findings revealed that GPS‐TILs offers strong prognostic value for overall survival (*p* < 0.0001, C‐index = 0.59), outperforming conventional TIL‐based measures and morphology‐based stratification approaches. These results highlight the potential of GPS‐TILs as a more objective and effective tool for improving patient risk stratification in LUAD. © 2025 The Author(s). *The Journal of Pathology* published by John Wiley & Sons Ltd on behalf of The Pathological Society of Great Britain and Ireland.

## Introduction

Lung cancer is a leading cause of cancer‐related mortality worldwide [1], with lung adenocarcinoma (LUAD) being the most prevalent subtype, representing ~40% of all non‐small cell lung cancer (NSCLC) cases [2]. While advances in targeted therapy and immunotherapy have improved outcomes for subsets of patients, mortality rates remain high [3]. Gaining a better understanding of the morphology and tumour immune microenvironment of such heterogonous cancers can assist in finding better predictive and prognostic biomarkers.

Histological grading of LUAD is based on architectural patterns (lepidic, acinar, papillary, solid, and micropapillary) [4]. The International Association for the Study of Lung Cancer (IASLC) Pathology Committee [5] proposed a grading system in which tumours are classified as well‐differentiated (Grade 1), moderately differentiated (Grade 2), or poorly differentiated (Grade 3), according to the predominant histological pattern and the proportion of high‐grade components (equal to or more than 20%). Higher grades are linked to more aggressive behaviour and lower survival rates. However, the classification of these histology patterns is highly subjective, exhibiting high inter‐ and intraobserver variability, limiting the ability of such grading systems to effectively stratify patients, particularly those in the intermediate group (G2) [6].

Tumour‐infiltrating lymphocytes (TILs), including those located in the tumour stroma, also known as stromal TILs (sTILs), have emerged as valuable prognostic indicators in several malignancies, including LUAD [7, 8]. Previous studies have shown that higher TIL densities are associated with improved survival and may predict response to immune checkpoint inhibitors [9]. Recent work has utilised machine‐learning approaches to objectively quantify TILs and sTILs from digitized whole‐slide images (WSIs), overcoming the subjectivity and reducing the labour cost of manual assessment [9, 10].

This study integrates two prognostic factors (TILs and growth patterns), hypothesizing that the tumour immune microenvironment may have different effects, depending on the underlying histology pattern. We utilised machine learning to produce a simple, objective, reproducible quantification of TILs, sTILs, and histological growth patterns in LUAD. Combining information on both immune response and tumour morphology, we address the gap in current prognosis biomarkers by developing GPS‐TILs, a histological growth‐pattern‐specific TILs measures capable of significantly stratifying patients into low‐ and high‐risk groups. Detailed experiments demonstrate that quantifying TILs within individual growth patterns yields more effective risk stratification than assessing them across the entire tumour.

## Materials and methods

### Dataset

A total of 493 formalin‐fixed paraffin‐embedded (FFPE) digitised WSIs of haematoxylin and eosin (H&E) stained section from 433 patients from The Cancer Genome Atlas (TCGA) [11] were utilised for this study. This dataset was retrieved from the TCGA‐LUAD cohort consisting of diagnostic slides of 478 patients diagnosed with LUAD. We excluded 14 invasive mucinous adenocarcinoma (IMA) cases; as IMA is a biologically distinct entity with a different risk profile and morphology to conventional LUAD [12]. We also performed a quality check excluding slides with major artifacts. For a detailed CONSORT diagram, see the supplementary material, Figure S1. The TCGA cohort was deidentified and publicly available, and participant consent had previously been obtained by The Cancer Genome Atlas Consortium.

### Clinicopathological features

The median follow‐up duration in our study cohort was 21 months, with a range of 0–238 months. Survival data were right‐censored by limiting the maximum survival analysis time to 60 months (5 years). The cohort consisted of a nearly equal number of female and male patients, with an average age of 64.92 years. The majority of patients were in stage I based on the TNM staging system. Grade information was manually extracted from patients' pathology reports. Table 1 summarises the clinicopathological features of the cohort used in this study.

**Table 1:** Summary of clinicopathological features in the study cohort

Variable	Subvariable	% or mean (SD)
Age (years)		64.92 (10.28)
Sex	Female	53.91%
	Male	46.09%
Stage	I	55.30%
	II	23.27%
	III	14.29%
	IV	5.53%
	Not given	1.61%
Smoking status	Smoker	70.97%
	Nonsmoker	29.03%
Grade	Grade 1	10.37%
	Grade 2	37.33%
	Grade 3	41.24%
	Not given	11.06%

### Nuclei detection and growth pattern classification

**Figure 1:**
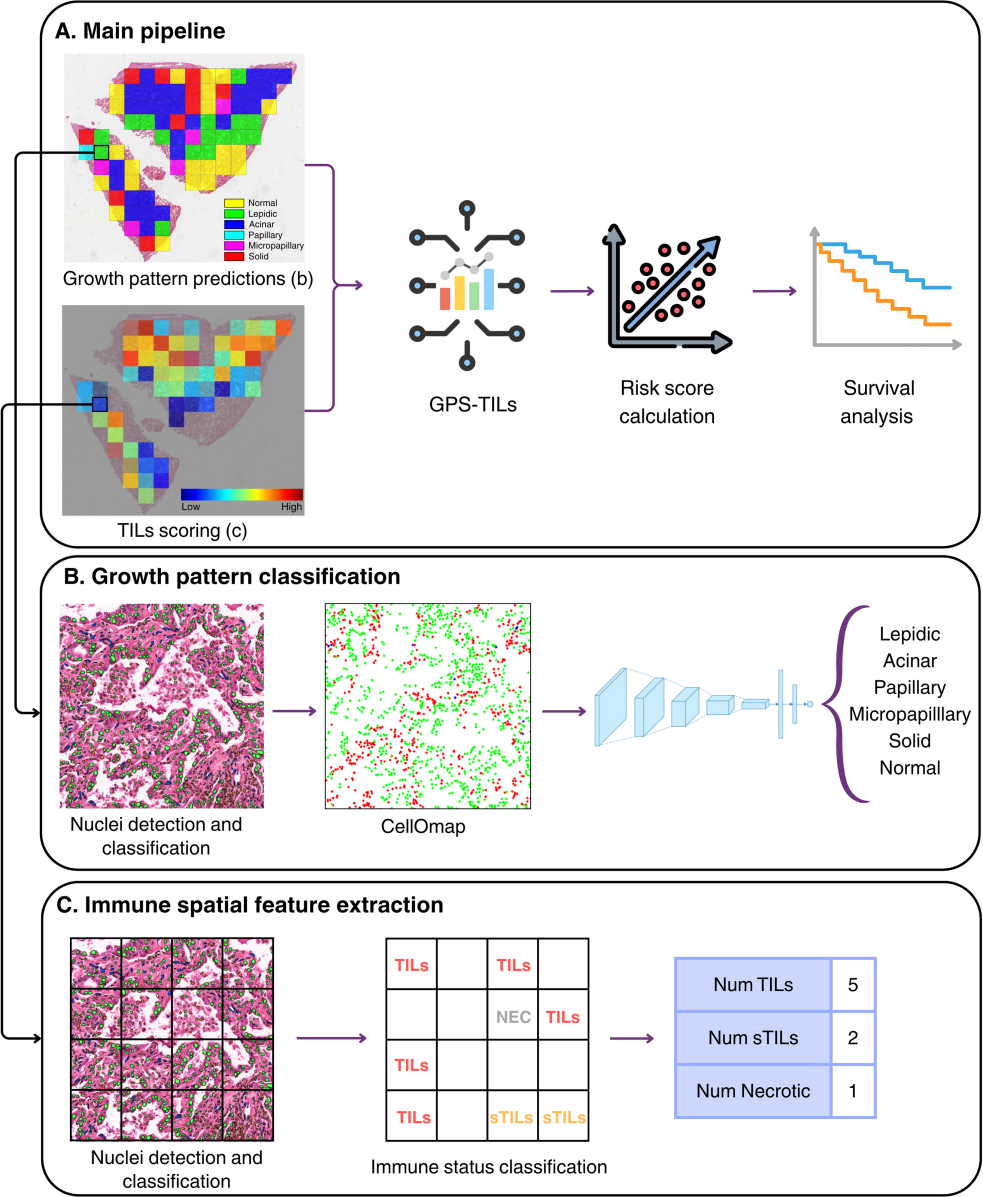
An overview of the analysis pipeline. (A) Main pipeline. (B) Using *CellOMaps* [4] for growth pattern classification. (C) Extraction of digital tumour‐immune spatial features.

Our analysis pipeline consisted of four main steps, as depicted in Figure 1. First, we detected and classified nuclei in WSIs at 20× magnification using the Hover‐Net model [13], which was trained on the PanNuke dataset [14]. To give an estimated accuracy measure of Hover‐Net on the used dataset, we sampled regions from random WSIs (containing an average of 184 nuclei/region). The predictions were manually validated by an expert pathologist, showing an accuracy of ~98.3%. We then used a previously developed in‐house model [15] to classify tissue regions into one of the five LUAD histological growth patterns: (lepidic, acinar, papillary, micropapillary, and solid). This model creates a compact representation of the tissue using cellular information denoted *CellOMaps*. A ResNet neural network model [16] was subsequently used to perform tissue classification of regions equivalent to 448 × 448, at 5× magnification. The model has an overall sensitivity of 0.70 and precision of 0.71, and can predict solid, lepidic, and acinar pattern with high precision (f1‐score of 0.93, 0.87, and 0.84, respectively) (see the supplementary material, Table S3 for per pattern accuracy and F1‐scores). Papillary and micropapillary patterns had lower but acceptable predictive precision (f1‐score of 0.59, and 0.67, respectively); given the high observer variability in these patterns.

### Digital tumour‐immune spatial features

To quantify immune activity in the patched regions using only counts of cell nuclei, we further subdivided each tissue patch into 16 equal nonoverlapping tiles, resulting in smaller localized areas of 112 × 112, at 5× magnification equivalent to 448 × 448, at 20× magnification. Inspired by the simple TILs quantification approach used by Chen *et al* [17] and Zhang *et al* [9], we classified each tile as TILs, sTILs, or neither by counting the nuclei in that tile. A tile was not considered if it contained less than 10 nuclei. A TILs tile was defined as one containing 20 or more neoplastic cells and at least 5 lymphocytes. Similarly, an sTILs tile was defined as one containing at least five lymphocytes, with stromal cells outnumbering neoplastic cells. Moreover, we flagged each tile as necrotic if it contained 15 or more necrotic cells. These numbers were set empirically. For each patch, we generated a three‐feature vector, representing the number of TILs, sTILs, and necrotic tiles (Figure 1C).

Finally, to obtain a single feature vector for each patient we calculated the minimum, maximum, mean, and SD of the TILs, sTILs, and necrotic counts in the entire tumour from all the WSIs associated with that patient, as well as in each histological pattern separately (see the supplementary material, Figure S7, which shows the distribution of TILs and sTILs in the different patterns). These features measure the presence, density, abundance, and dispersion of the cells in the study, respectively. Counts associated with a micropapillary pattern were excluded from analysis due to its limited representation in the cohort, and the additional noise they introduced when included (supplementary material, Figure S10). See the supplementary material Table S1 for a detailed description of the case features.

### Survival analysis

We used overall survival (OS) as the primary endpoint in our survival analysis, with patients right‐censored at 5 years. We also examined additional endpoints such as disease‐specific survival (DSS), and progression‐free interval (PFI). Python 3.11 (Python Software Foundation, https://www.python.org/) was used throughout the study, including model development and statistical analyses.

We employed three sets of features to show their prognostic value in LUAD: (1) histological growth pattern percentages, (2) the presence, density, abundance, and dispersion of TILs, sTILs, and necrosis area in the entire tumour, and (3) measured the same features in each histological pattern separately, which we defined as our growth‐pattern‐specific TILs biomarker (GPS‐TILs). For each experiment, we trained a Cox proportional hazards (Cox PH) model using two‐fold cross‐validation to predict patient risk scores. Harrell's concordance index (C‐index) [18] was used to evaluate the model's risk score predictions on the validation set by measuring the proportion of concordant pairs (where predicted risk scores correctly rank survival times) among all possible pairs.

To evaluate the prognostic value of risk scores obtained from the proposed digital features, the median of the discovery set was used as a cutoff value to stratify patients into high‐ and low‐risk groups. Individuals with scores at or below the cutoff value were assigned to the low‐risk group, while those above were classified as high‐risk. We plotted the Kaplan–Meier (KM) survival curves of the entire cohort by aggregating the results of the validation set from all cross‐validation folds. We performed a permutation test, with 1,000 permutations, on the aggregated predictions to assess the significance of the proposed digital features in stratifying patients.

## Results

We quantified the prognostic value of LUAD grading and showed how stratifying TILs by histological patterns improved patient stratification. Two‐fold cross‐validation was used to show the generalisation of the proposed predictive features.

### The prognostic value of LUAD grading

**Figure 2:**
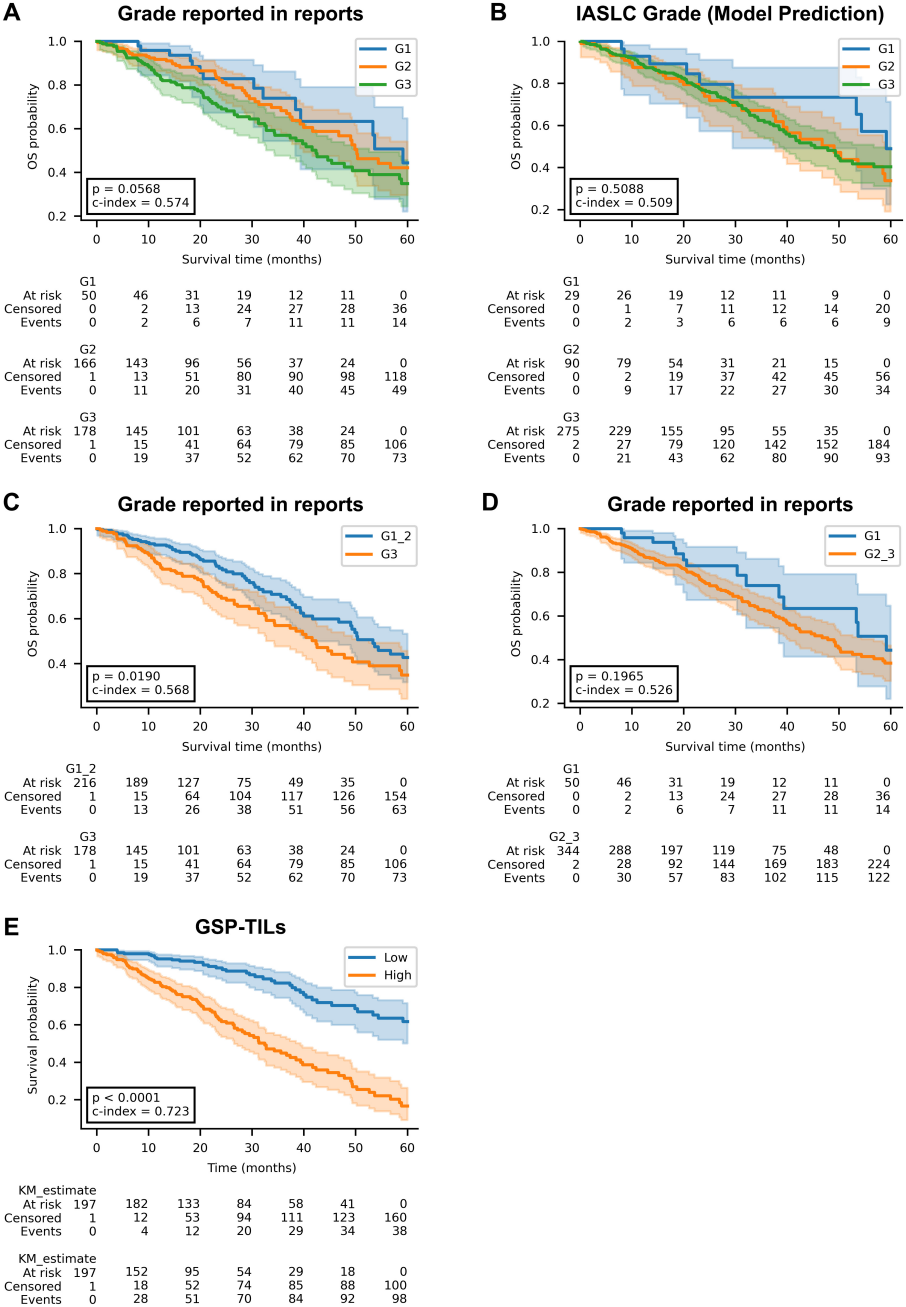
Kaplan–Meier (KM) survival curves for (A) Grades extracted from patient reports.(Grade 1 and Grade 3 are separable (*p* = 0.0256)). (B) Grades based on the International Association for the Study of Lung Cancer (IASLC) grading system calculated from the growth pattern classifier predictions. (C) Grade 1 and Grade 2 versus Grade 3. (D) Grade 1 versus Grade 2 and Grade 3. (E) Risk scores from the proposed GPS‐TILs marker.

In current clinical practice, the grading of LUAD tumours is primarily based on subjective assessment and quantification of growth patterns observed in histological sections [4]. To assess the prognostic value of such grading, we plotted KM survival curves based on the assigned grades for patients with available grade information in their reports (*n* = 394) (Figure 2A). We then used the model's predicted growth patterns for the same patients and applied the IASLC Pathology Committee grading system [5], which defines grade according to the predominant pattern and the proportion of high‐grade components, to assign grades and generate corresponding KM curves (Figure 2B). Both grading approaches demonstrated comparable patient stratification; however, differences between grade groups were not statistically significant (*p* = 0.0568 and *p* = 0.5088). Patients with Grade 1 and Grade 3 tumours could be clearly stratified by outcome (*p* = 0.0256). However, Grade 2 tumours did not separate well from either Grade 1 or Grade 3, reducing the overall prognostic utility of the three‐tier system. This may be due to interobserver variability and the biological heterogeneity within intermediate‐grade tumours. Similarly, no significant survival stratification was observed when using the dominant growth pattern alone (supplementary material, Figure S2). This highlights the need for further investigation into the prognostic utility of growth patterns beyond mere quantitative assessment.

We further combined Grade 1 and Grade 2 into a low‐grade group, and Grade 3 into a high‐grade group, which improved stratification (*p* = 0.019; Figure 2C). However, the C‐index remained low, at 0.568. Combining Grade 2 and Grade 3 into high grade and Grade 1 as low‐grade group did not show good stratification (*p* = 0.1965; Figure 2D). In contrast, the proposed GPS‐TILs marker demonstrated significantly improved stratification (*p* < 0.0001; C‐index = 0.723) as shown in Figure 2E. This finding demonstrates the superior ability of the GPS‐TILs score to rank patient survival compared to all grading‐based approaches.

### Stratifying TILs and sTILs based on histological patterns

We investigated the performance of various TILs, sTILs, and necrotic area quantification approaches in survival prediction using the Cox PH model. As shown in Table 2, the best performing feature set was our proposed GPS‐TILs marker, which quantified TILs, sTILs, and necrotic area in each histological growth pattern separately (C‐index = 0.59). Although TILs and sTILs only provided good survival prediction (C‐index = 0.56), the performance was improved when necrotic area features were added. The GPS‐TILs marker enabled significant stratification of patients into low‐ and high‐risk groups (*p* < 0.0001), outperforming the same measures calculated on the entire tumour area (C‐index = 0.52; *p* = 0.367). The addition of pattern percentages to the overall measures improved the predictions but did not perform as well as the growth‐pattern‐specific measures. Pattern percentages did not add any significant improvement when combined with the stratified measures. Moreover, combining grade information with the GPS‐TILs marker did not add show significant improvement (C‐index = 0.60; *p* = 0.002). The GPS‐TILs marker yielded similar stratification in other survival endpoints, such as DSS (*p* = 0.009), and PFI (*p* = 0.013) (supplementary material, Figure S3).

**Table 2:** Cross‐validation results for overall survival using the Cox proportional hazards model

Features	Validation set C‐index (mean ± SD)
Overall TILs, sTILs and necrosis	0.523 ± 0.038
Overall TILs, sTILs and necrosis and pattern percentages	0.548 ± 0.027
Growth pattern TILs, sTILs	0.562 ± 0.012
Growth pattern TILs, sTILs, and necrosis (GPS‐TILs)	0.594 ± 0.024
GPS‐TILs and pattern percentages	0.598 ± 0.015
GPS‐TILs and grade	0.596 ± 0.033

GPS‐TILs, growth‐pattern‐specific tumour infiltrating lymphocytes; TILs, tumour infiltrating lymphocytes.

**Figure 3:**
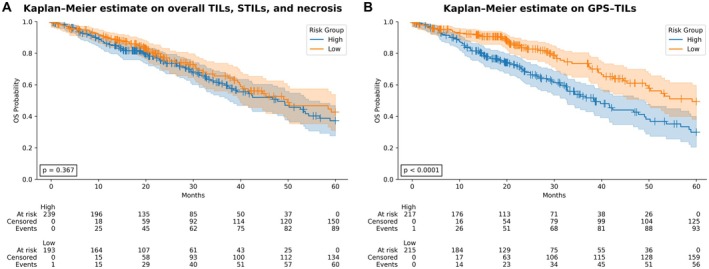
Kaplan–Meier (KM) survival curves for cross‐validation results using (A) TILs, sTILs, and necrotic area in the entire tumour, and (B) TILs, sTILs and necrotic area in different growth patterns.

Figure 3 illustrates the KM curves of the different feature sets of the entire cohort by combining the validation sets from the cross‐validation folds. Reported *p* values were calculated using a permutation test, with 1,000 permutations.

### Adjustment for clinicopathological factors

**Figure 4:**
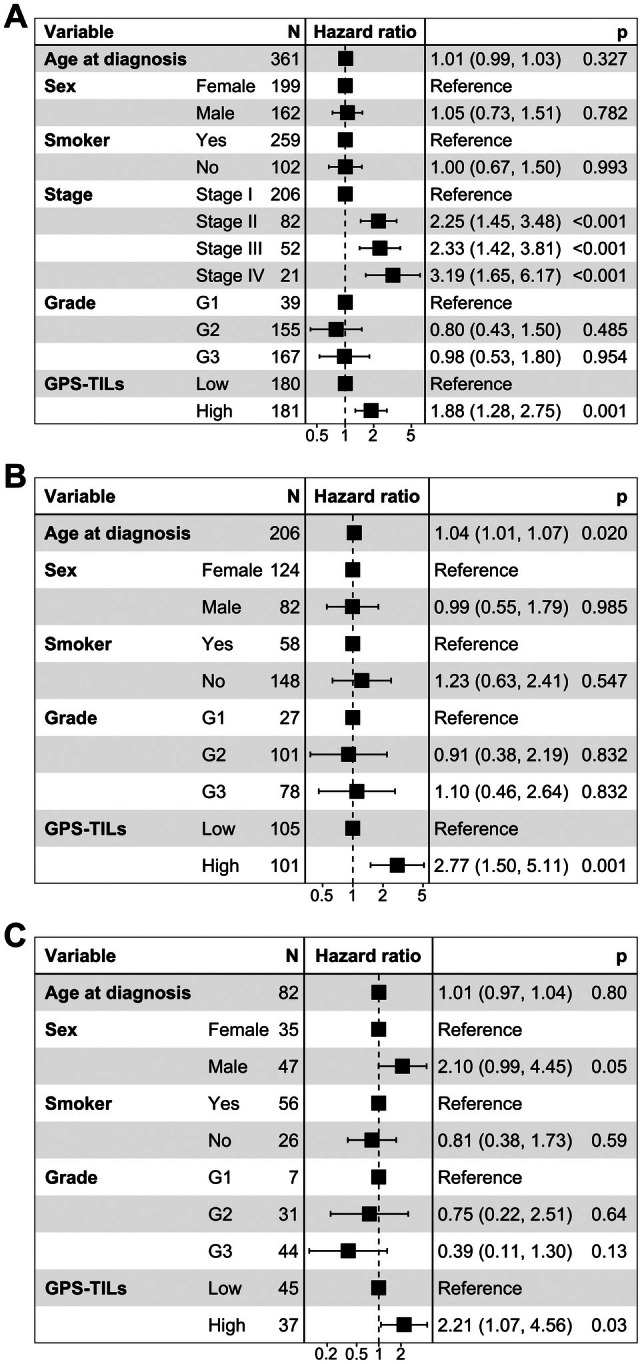
Forest plot showing HR with 95% confidence intervals (CI) and *p* values (of the log‐rank test) GPS‐TILs marker when adjusted for other clinicopathological variables on OS of (A) all patients, (B) Stage I patients, (C) Stage II patients.

The prognostic value of the proposed GPS‐TILs marker was further evaluated using a multivariate analysis adjusted for other clinicopathological factors. Figure 4A shows a forest plot from the multivariate Cox PH regression model for OS, displaying hazard ratios (HRs) and 95% confidence intervals (CIs). The GPS‐TILs marker demonstrated independent prognostic significance (*p* = 0.002; HR = 1.85; CI: 1.26, 2.70). Stage was also found to be significant, with increasing stages showing a statistically significant increase in hazard. Other clinicopathological features including age, sex, smoking status, and grade did not show statistically significant independent prognostic value. Further analysis of stage I (Figure 4B), and stage II (Figure 4C) patients showed that GPS‐TILs can further significantly stratify the patients in a single stage. These results suggest that the GPS‐TILs marker has additive value over other clinicopathological features, including grade and stage (supplementary material, Figures S8 and S9).

### Univariate and multivariate analysis

**Figure 5:**
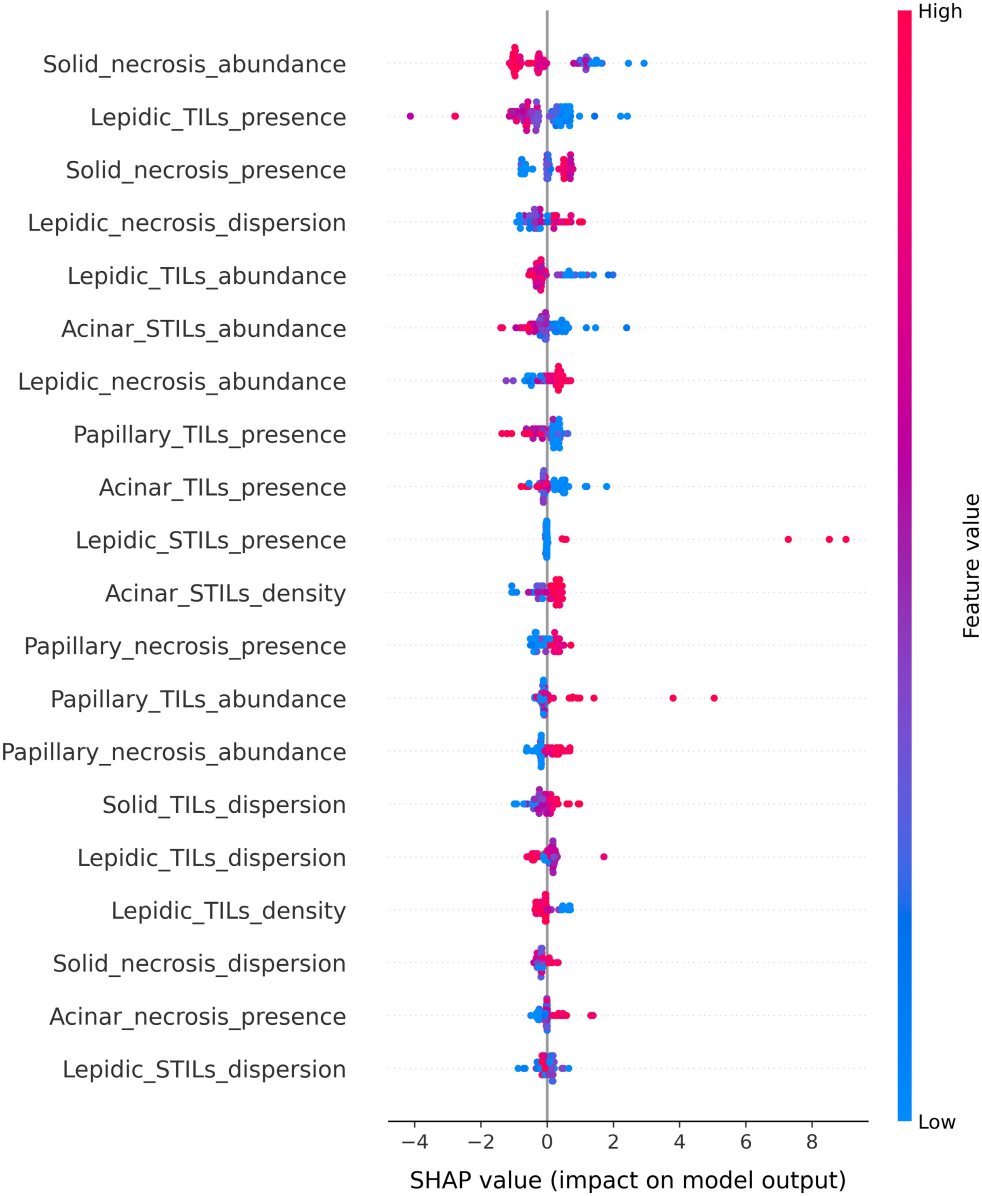
SHAP summary plot visualizing the top 20 important features contributing to the model's predicted risk score. The presence and abundance of necrosis in the solid pattern, the presence of sTILs in the lepidic pattern, and the abundance and density of sTILs in the acinar pattern increase the predicted risk. On the other hand, the presence of TILs in all patterns and their abundance in the lepidic pattern decrease the predicted risk.

Both univariate and multivariate analyses were employed to evaluate the relationship between risk factors and survival outcomes using the Cox PH model. As shown in the supplementary material, Figure S4, univariate analysis showed that no single feature demonstrated statistically significant association with survival when considered in isolation, as reflected by wide confidence intervals and insignificant *p* values. However, in the multivariate analysis (supplementary material, Figure S5) some features emerged as potentially informative, such as: papillary_sTILs_dispersion (*p* = 0.025), solid_necrosis_density (*p* = 0.014), solid_TILs_density (*p* = 0.038), and Lepidic_sTILs_presence (*p* = 0.072), indicating that these features may have predictive value only in the context of the broader feature set. While most variables did not reach statistical significance individually, their inclusion in the multivariate model contributed to capturing the multifactorial nature of tumour biology and prognosis. To further understand the influence of the different features on the predicted risk score, we performed SHAP (SHapley Additive exPlanations) analysis [19]. SHAP values were used to quantify the contribution of each feature to a model's prediction, indicating both the direction (positive or negative influence) and magnitude of effect. Positive SHAP values indicate that a feature increases the predicted outcome, while negative values suggest a decrease. This allows for a transparent understanding of both the direction and magnitude of each feature's influence, facilitating interpretability and trust in model predictions. The results shown in Figure 5 indicate that the presence and abundance of necrosis in the solid pattern was a major contributor to the predicted risk score. The presence of TILs in all patterns and their abundance in the lepidic pattern was a major factor in decreasing the predicted risk. Other major features that increase the predicted risk included the presence of sTILs in the lepidic pattern and the abundance and density of sTILs in the acinar pattern.

### Risk score is independent from clinical and demographic variables

To evaluate the independence of our proposed GPS‐TILs risk score, we tested its association with key clinicopathological features that could serve as potential confounding factors. These included sex, age, ethnicity, smoking history, and growth pattern percentages. We also assessed its association with AJCC staging components. Continuous variables were tested using Spearman correlations; categorical variables were assessed using Mann–Whitney U‐tests, or ANOVA tests as appropriate. Effect sizes (e.g. Cohen's d) were calculated for binary group comparisons to assess the strength of association. All tested potential confounding factors, including sex (Cohen's d = −0.19), smoking history, and growth pattern percentages, showed either nonsignificant or negligible associations with the risk score (*p* > 0.05 or small effect sizes). On the other hand, risk scores were statistically significant among different stages (*p* = 0.0022), indicating a potential relationship with stage (see the supplementary material, Table S2 for detailed analysis).

## Discussion

TILs have been extensively studied as prognostic biomarkers across various cancer types, including LUAD. Their abundance is frequently associated with favourable clinical outcomes, reflecting an active anti‐tumour immune response [20, 21]. Several studies have leveraged deep‐learning‐based approaches to automate the quantification of TILs [9], demonstrating comparable performance to manual assessment by pathologists. These findings highlight the potential of artificial intelligence to enhance diagnostic efficiency and reduce pathologists' workload. Histological growth patterns are another prognostic factor that are used to Grade LUAD tumours [4]. In this study we hypothesized that the prognostic impact of TILs might vary according to the histological pattern in which they are situated. Our findings from a large, multicentre LUAD cohort empirically support this hypothesis.

To the best of our knowledge, this study is among the first to quantitively assess TILs within LUAD growth patterns using AI‐driven methods. We show that quantifying TILs in each growth pattern separately improves patient stratification. We define several features that describe the overall distribution of lymphocytes inside the tumour area and in the stroma within or adjacent to the tumour. These descriptive features measure the presence, density, abundance, and dispersion of the lymphocytes in the tumour microenvironment.

### TILs and sTILs in the lepidic growth (observational finding)

**Figure 6:**
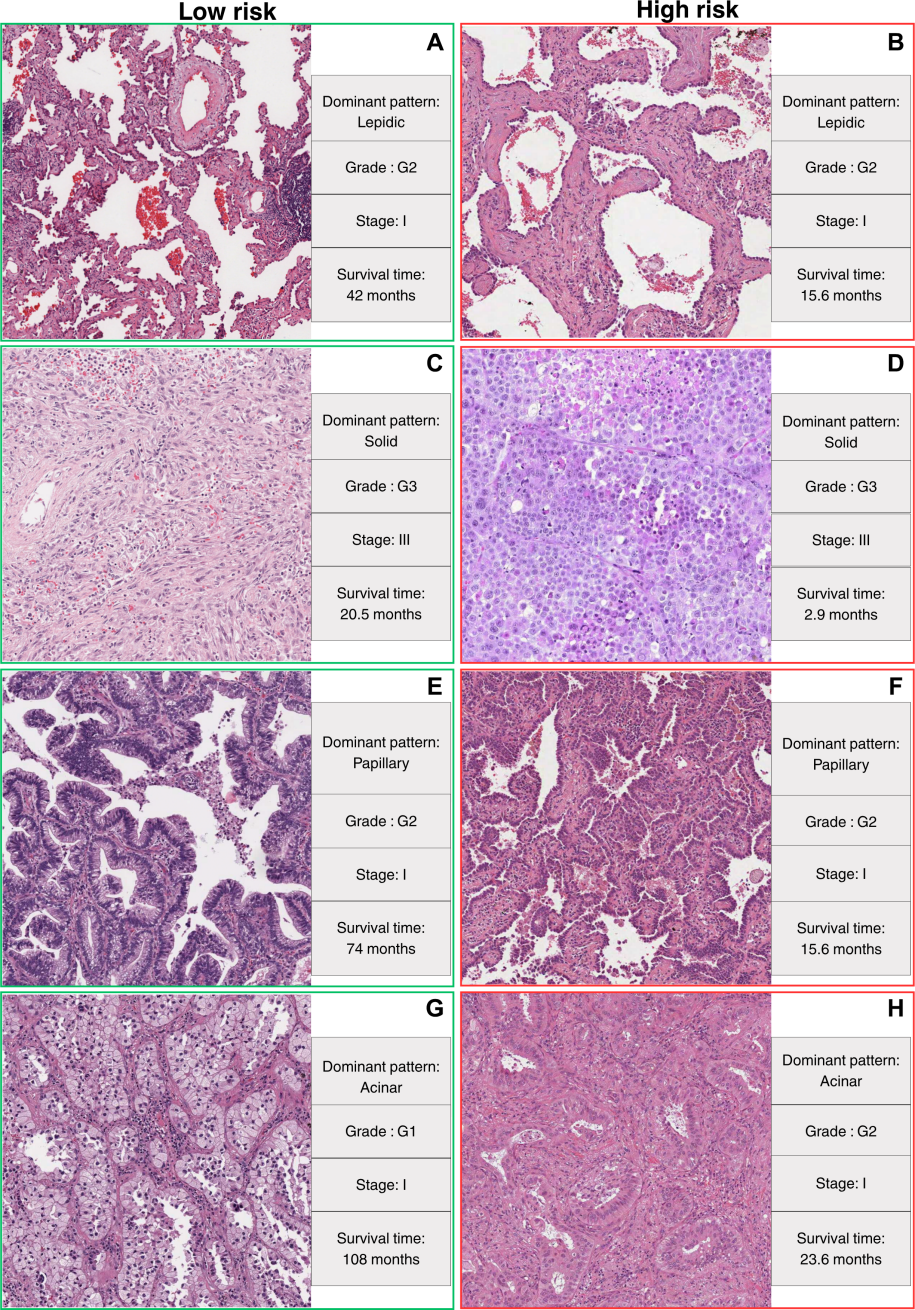
Samples from WSIs with good and poor outcomes. (A) A lepidic pattern from a case predicted as low‐risk, with high TILs abundance and low sTILs. (B) A lepidic pattern from a case predicted as high‐risk, with the presence of sTILs and low TILs. (C) A solid pattern from a case predicted as low‐risk, with high sTILs and TILs. (D) A solid pattern from a case predicted as high‐risk, with few sTILs and TILs. (E) A papillary pattern from a case predicted as low‐risk, with high sTILs dispersion. (F) A papillary pattern from a case predicted as high‐risk, with low sTILs dispersion. (G) An acinar pattern from a case predicted as low‐risk, having low sTILs. (H) An acinar pattern from a case predicted as high‐risk, with less abundant sTILs. Grades were obtained from the original pathology reports.

Further analysis shows that the abundance of TILs is important in all patterns, especially in the areas with lepidic growth. In lepidic‐predominant cases, prognosis was more favourable overall, but we noted that adverse outcomes were more frequent when lepidic areas were adjacent to sTIL‐rich stroma or transitioning to invasive components. This highlights the potential prognostic relevance of the lepidic‐invasive interface, although we emphasise that this was an observational finding and no quantitative testing was performed. Figure 6A shows a lepidic pattern from a patient with favourable outcome where we can observe TILs enrichment. On the other hand, Figure 6B shows a lepidic pattern in a patient with unfavourable outcome.

### Immune‐rich intratumoral stroma within the solid pattern is associated with better prognosis

The solid pattern is associated with advanced tumour stage and poor prognosis [5]. In our cohort, we observed that the presence of immune‐rich intratumoral stroma was associated with a more favourable prognosis. Such findings are consistent with the concept of an immune‐active tumour microenvironment, which may be amenable to immunotherapeutic intervention [20]. We emphasize that this was an observational finding without quantitative testing. Figure 6C,D shows a solid pattern from patients with a solid dominant pattern having good and poor prognosis, respectively. The difference in the amount of immune enriched stroma is clearly evident.

### Low sTIL dispersion relates to architectural complexity in a papillary pattern

In the papillary pattern, we observed that lower dispersion of sTILs appeared to correspond to regions with compact and densely arranged papillary structures. Previous studies [22] have reported that papillary adenocarcinoma can show variable architectural complexity and includes morphologically distinct subtypes, where densely packed or complex papillary structures, often disrupting alveolar architecture, are associated with a more aggressive clinical course. In contrast, well‐formed, delicate papillae retaining some of the underlying alveolar architecture appear to be linked to more indolent behaviour. While our findings suggest a potential association between sTILs dispersion and papillary morphology, these are exploratory visual observations, and further validation is required to confirm this correlation. Figure 6E,F shows samples of papillary patterns from patients with good and poor prognosis, respectively. In the case with favourable outcome, we can observe that the papillae are thinner and well separated compared to the case with poor outcome.

### Stromal separation in acinar glands degrades prognosis (observational finding)

In the acinar pattern, our analysis revealed that increased stromal separation between glandular structures was more common in high‐risk cases. This raises the possibility that a more expansive stromal component may reflect tumour progression or desmoplastic response, potentially indicating a more invasive microenvironment. While stromal regions often host immune infiltrates, in our dataset this did not always translate to favourable outcomes, suggesting that the spatial distribution of stroma may influence prognosis. These findings are preliminary and warrant further investigation to understand the biological significance of stromal expansion in acinar type LUAD. Figure 6G,H shows samples of acinar pattern from patients with good and poor prognosis, respectively.

### Presence of necrosis in solid tumours is associated with poor prognosis

Necrosis within solid tumours is a well‐recognised adverse prognostic feature, reflecting tumour hypoxia and rapid growth [23, 24]. Necrosis often results due to inadequate blood supply as the tumour rapidly grows. The resulting hypoxia stabilises HIF‐1α, promoting angiogenesis, metabolic reprogramming, and resistance to radiotherapy and chemotherapy [25]. Our findings are in line with these observations, highlighting necrosis as a marker of poor prognosis in LUAD.

### Risk groups express differences in immune gene signature

We further investigated the differences in immune features, measured from RNA‐seq data, reported in Thorsson *et al* [1, 26] between the low‐risk and high‐risk groups, as stratified by the proposed GPS‐TILs marker. We conducted Mann–Whitney *U*‐tests, with *p* values adjusted for multiple comparisons using the Benjamini–Hochberg correction. As shown in the supplementary material, Figure S6A, the low‐risk group demonstrated an elevated lymphocyte infiltration signature score, which aligns with our histological nuclei counts score. The analysis of immune features also showed a significant increase in proliferation in the high‐risk group. High proliferation reflects enhanced cellular division and tumour growth, often associated with aggressive tumours and poor clinical outcomes [27]. In contrast, macrophage regulation scores were significantly higher in the low‐risk group, suggesting a potentially more balanced or immunologically active tumour microenvironment.

### Low‐risk LUAD patients show a richer and more diverse immune T‐cell receptor repertoire

Low‐risk LUAD patients appear to exhibit a significantly more abundant and diverse repertoire of immune T‐cell receptors (TCRs) within their tumour microenvironment (supplementary material, Figure S6C,D). To determine whether high TCR repertoire diversity was mainly due to lymphocyte abundance, we normalized the TCR Shannon diversity by log‐transformed TILs abundance. Low‐risk patients retained significantly higher normalized TCR Shannon diversity compared to high‐risk patients (Mann–Whitney *U* = 0.0001414; *p* = 0.001157) (supplementary material, Figure S11), indicating that the observed diversity difference between groups is not merely driven by the overall abundance of TILs. This diversity suggests a more robust and adaptable immune response, as a richer TCR repertoire enables the immune system to recognize and target a broader array of tumour‐associated antigens [28, 29]. Importantly, a diverse TCR repertoire has been closely associated with better responses to immunotherapies, such as immune checkpoint inhibitors. This wide variety of T‐cell clones allow the detection of multiple tumour‐specific neoantigens, increasing the immune system's ability to recognise and attack cancer cells [30, 31].

### High‐risk LUAD patients express higher levels of Th2 cells

High‐risk LUAD patients express significantly higher levels of Th2 cells (supplementary material, Figure S6B), which are known to contribute to an immunosuppressive tumour environment. Th2 cells typically secrete cytokines, which can inhibit the activation and function of cytotoxic T‐lymphocytes and other anti‐tumour immune cells [32]. The enrichment of Th2 cells is an indicator of potential resistance to immunotherapy, and thus resulting in poorer survival [33].

### High‐risk LUAD patients show increased mitotic activity

Using the approach by Jahanifar *et al* [34] for mitosis detection and quantification, we analysed the number of mitotic figures in both low‐ and high‐risk groups stratified by the proposed GPS‐TILs marker (supplementary material, Figure S6E). High‐risk LUAD patients exhibit significantly increased mitotic activity (*p* < 0.01), with a notably higher frequency of atypical mitotic figures (*p* < 0.001), compared to low‐risk patients. High mitotic activity, especially atypical mitoses, have been linked to chromosomal instability, aiding tumour progression [35]. The mitotic index, which is the percentage of mitosis cells, has been shown to be an independent predictor of outcome in various cancer types [36, 37].

## Conclusion

In summary, we present a novel machine‐learning pipeline capable of stratifying patients using standard H&E images. We detected histological growth patterns present in a slide and quantified TILs and sTILs using only nuclei counts. This study highlights the current limitations in the grading of LUAD cancers and proposes an improved approach that utilises the histological patterns in such tumours. We demonstrate that quantifying TILs and sTILs in each histological pattern separately enhances patient stratification. These findings may provide improved insights into the behaviour of LUAD tumours and potentially assist with treatment planning.

Furthermore, we analysed the significant differences in the tumour immune microenvironment and in the immune gene signature across risk groups, which highlight their distinct biological profiles. However, some qualitive observations were based on expert review of a selected subset of cases and may therefore be subject to interpretation bias. Developing machine‐learning methods that can quantitively evaluate these observations open new avenues of research that may assist in generating quantifiable and objective findings. Additional analysis on larger and multicentric cohorts are essential to validate the generalisation of such findings. Finally, as this study relies on nuclei counts to determine TILs, STILs, and necrosis, integrating the knowledge of histological components (such as segmented tumour, stroma, and necrotic area) may add more precision. Moreover, integrating data on patient therapy could provide valuable insights for guiding targeted therapeutic strategies.

## Author contributions statement

AAR, SEAR and NMR conceptualised the study. AAR wrote the code, conducted and evaluated the experiments, and wrote the initial draft of the article. AA provided expert clinical and pathology input, critically reviewed the experimental outputs, and contributed to the interpretation of findings. SR and NMR supervised the development of the methodology. All authors read, modified and approved the final article.

## Supporting information


**Figure S1.** CONSORT diagram showing the criteria used to include and exclude cases in our study
**Figure S2**. Kaplan–Meier (KM) survival curves stratified by the dominant histological pattern
**Figure S3**. Kaplan–Meier (KM) survival curves from cross‐validation results using TILs, sTILs, and necrotic area across different growth patterns for two different endpoints
**Figure S4**. Forest plot for univariate analysis using the Cox proportional hazards model and overall survival as end point for the proposed features
**Figure S5**. Forest plots for multivariate analysis using the Cox proportional hazards model and overall survival as end point for the proposed features
**Figure S6**. Boxplots showing the distribution of different tumour immune microenvironment features across predicted low‐risk and high‐risk groups
**Figure S7**. Violin plots showing the distribution of TILs and sTILs counts in the different growth patterns in the entire cohort
**Figure S8**. Kaplan–Meier curves for GPS‐TILs marker stratified by the dominant pattern
**Figure S9**. Kaplan–Meier curves for GPS‐TILs marker stratified by grade
**Figure S10**. Kaplan–Meier (KM) survival curves for cross‐validation results using: TILs, sTILs and necrotic area in different growth patterns (including micropapillary), C‐index = 0.57
**Figure S11**. Boxplot showing the distribution of the TCR Shannon normalized by log‐transformed TILs abundance for predicted low‐risk and high‐risk groups
**Table S1**. Description of the features used to derive the digital biomarker
**Table S2**. Risk score association with key clinicopathological features
**Table S3**. Average and standard deviation of per class accuracy and F1 for *CellOMaps* on TCGA‐LUAD using patient‐level cross‐validation

## Data Availability

The dataset TCGA‐LUAD is publicly available on The Cancer Genome Atlas Program. We have released the code for reproducing results along with the grades extracted from TCGA‐LUAD reports at the following URL: https://github.com/Arwa-AlRubaian/GPS_TILs. We collected a collection of representative tiles supporting the findings of this article in the following URL: https://zenodo.org/records/16761191.
