# Supplementary material for: Novel growth pattern‐specific digital marker of TILs improves stratification of lung adenocarcinoma patients
Source: J Pathol. 2025 Nov 15;268(2):164–75. doi: 10.1002/path.6498 (PMC12805620; doi:10.1002/path.6498)
Supplement: Supplementary file 1 — Figure S1. CONSORT diagram showing the criteria used to include and exclude cases in our study Figure S2. Kaplan–Meier (KM) survival curves stratified by the dominant histological pattern Figure S3. Kaplan–Meier (KM) survival curves from cross‐validation results using TILs, sTILs, and necrotic area across different growth patterns for two different endpoints Figure S4. Forest plot for univariate analysis using the Cox proportional hazards model and overall survival as end point for the proposed features Figure S5. Forest plots for multivariate analysis using the Cox proportional hazards model and overall survival as end point for the proposed features Figure S6. Boxplots showing the distribution of different tumour immune microenvironment features across predicted low‐risk and high‐risk groups Figure S7. Violin plots showing the distribution of TILs and sTILs counts in the different growth patterns in the entire cohort Figure S8. Kaplan–Meier curves for GPS‐TILs marker stratified by the dominant pattern Figure S9. Kaplan–Meier curves for GPS‐TILs marker stratified by grade Figure S10. Kaplan–Meier (KM) survival curves for cross‐validation results using: TILs, sTILs and necrotic area in different growth patterns (including micropapillary), C‐index = 0.57 Figure S11. Boxplot showing the distribution of the TCR Shannon normalized by log‐transformed TILs abundance for predicted low‐risk and high‐risk groups Table S1. Description of the features used to derive the digital biomarker Table S2. Risk score association with key clinicopathological features Table S3. Average and standard deviation of per class accuracy and F1 for CellOMaps on TCGA‐LUAD using patient‐level cross‐validation [file PATH-268-164-s001.docx]

**Novel growth pattern-specific digital marker of TILs improves stratification of lung adenocarcinoma patients**

A AlRuabain, A Azam *et al. J Pathol* <https://doi.org/10.1002/path.6498>

**Supplementary Figures S1–S11**

**Supplementary Tables S1–S3**


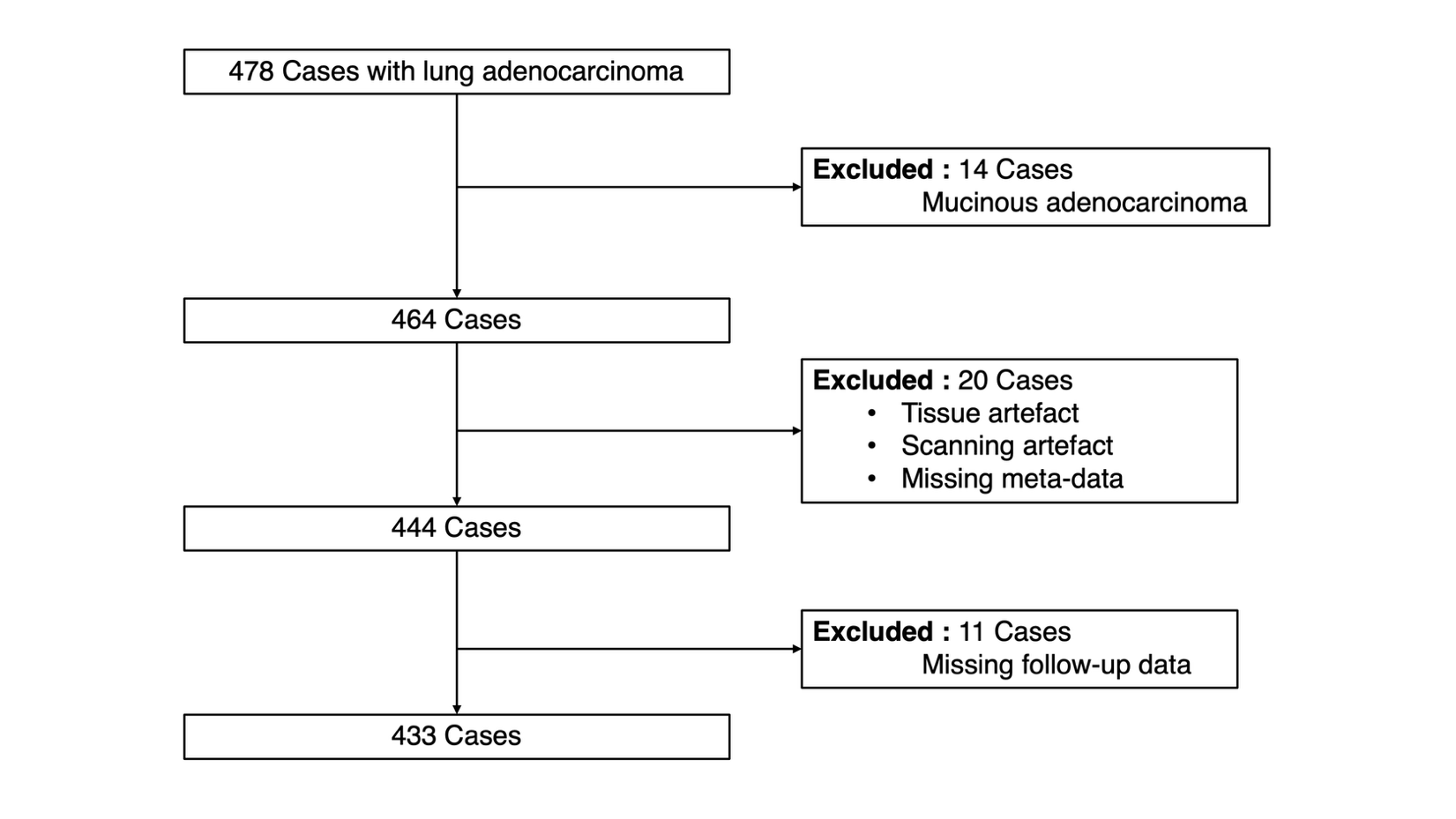


**Figure S1**. **CONSORT diagram showing the criteria used to include and exclude cases in our study.**


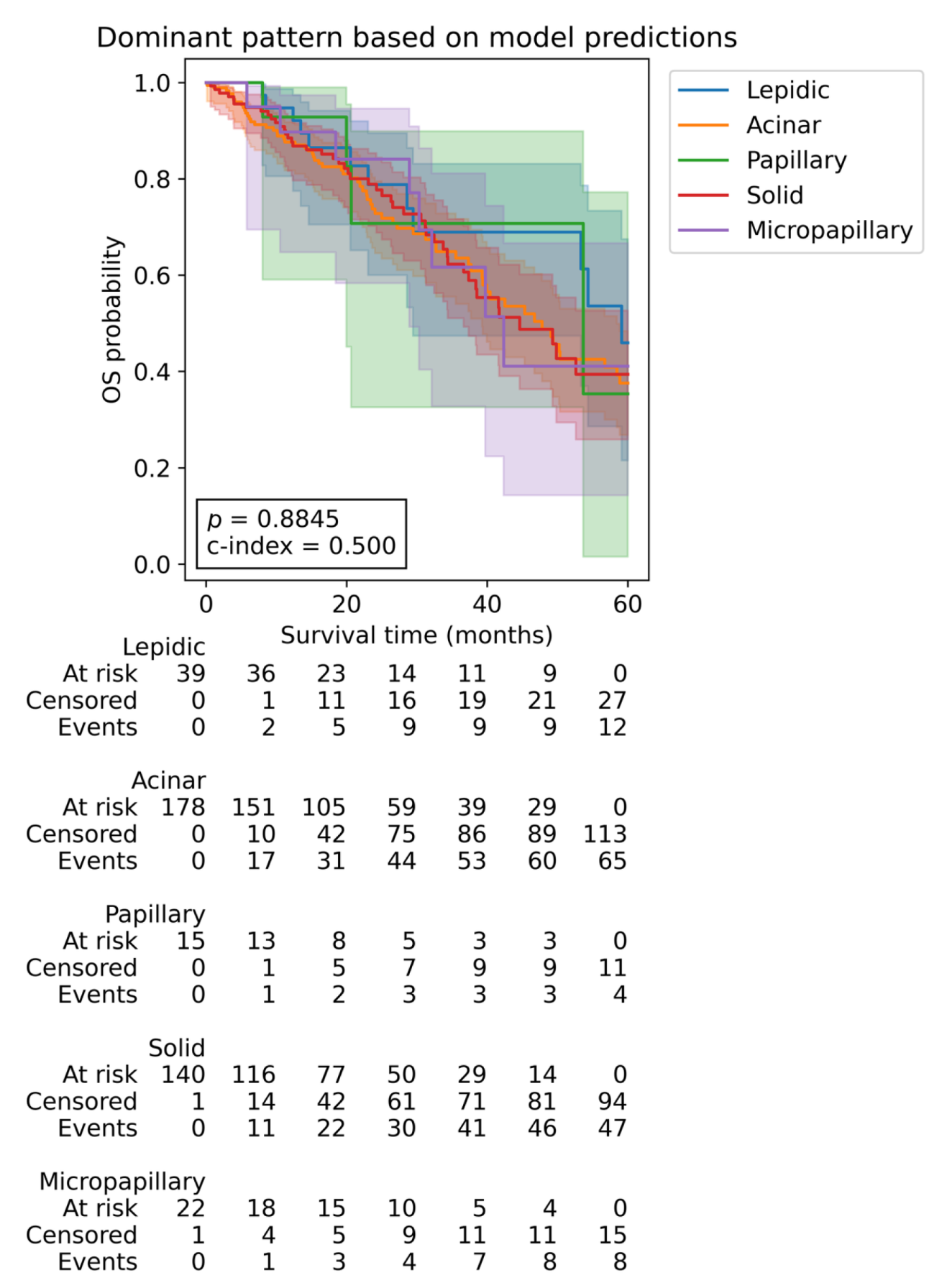


**Figure S2**. **Kaplan–Meier (KM) survival curves stratified by the dominant histological pattern.**


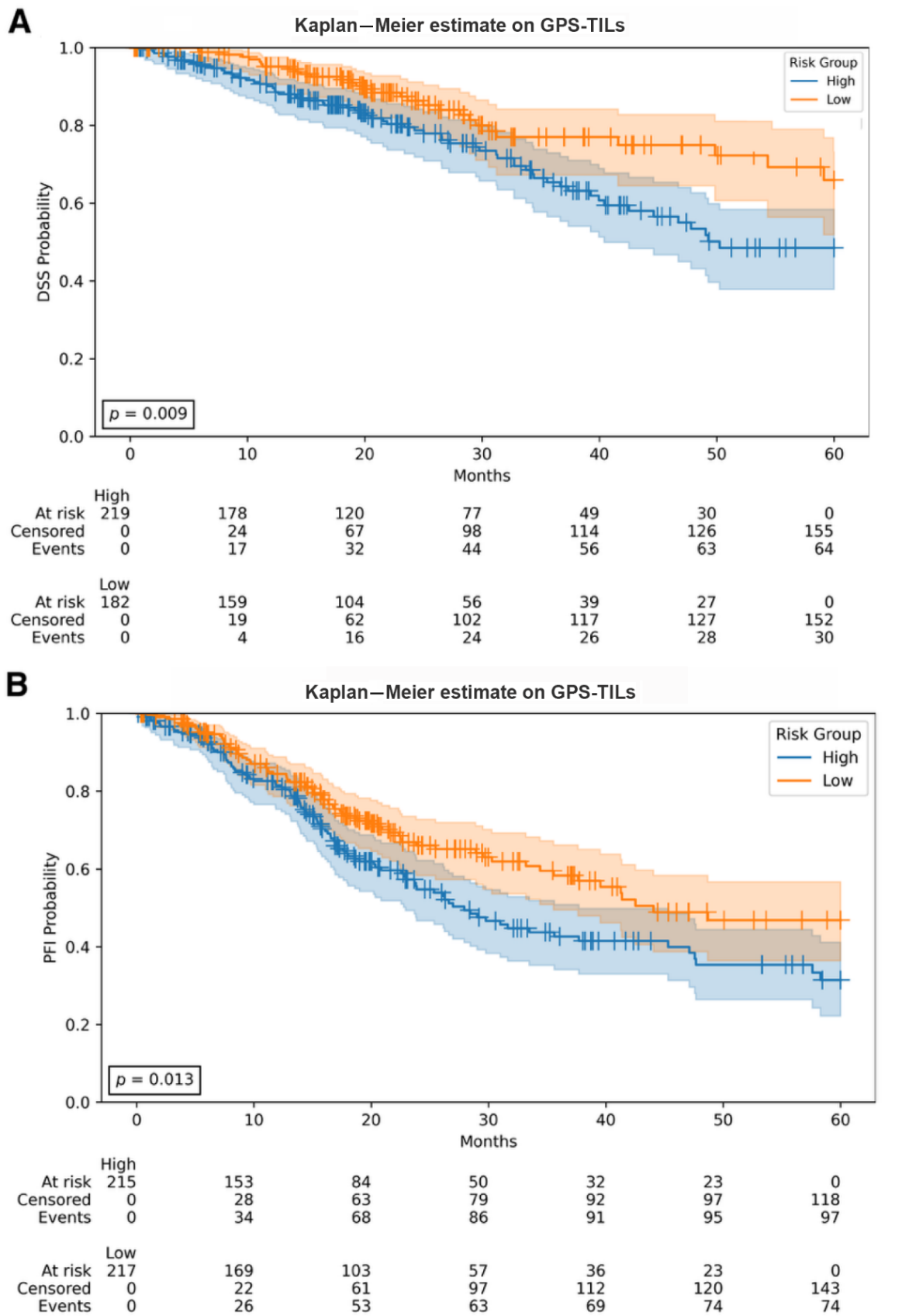


**Figure S3.** **Kaplan–Meier (KM) survival curves from cross-validation results using TILs, sTILs, and necrotic area across different growth patterns for two different endpoints.** (A) Disease-specific survival (DSS), (B) Progression-free interval (PFI).


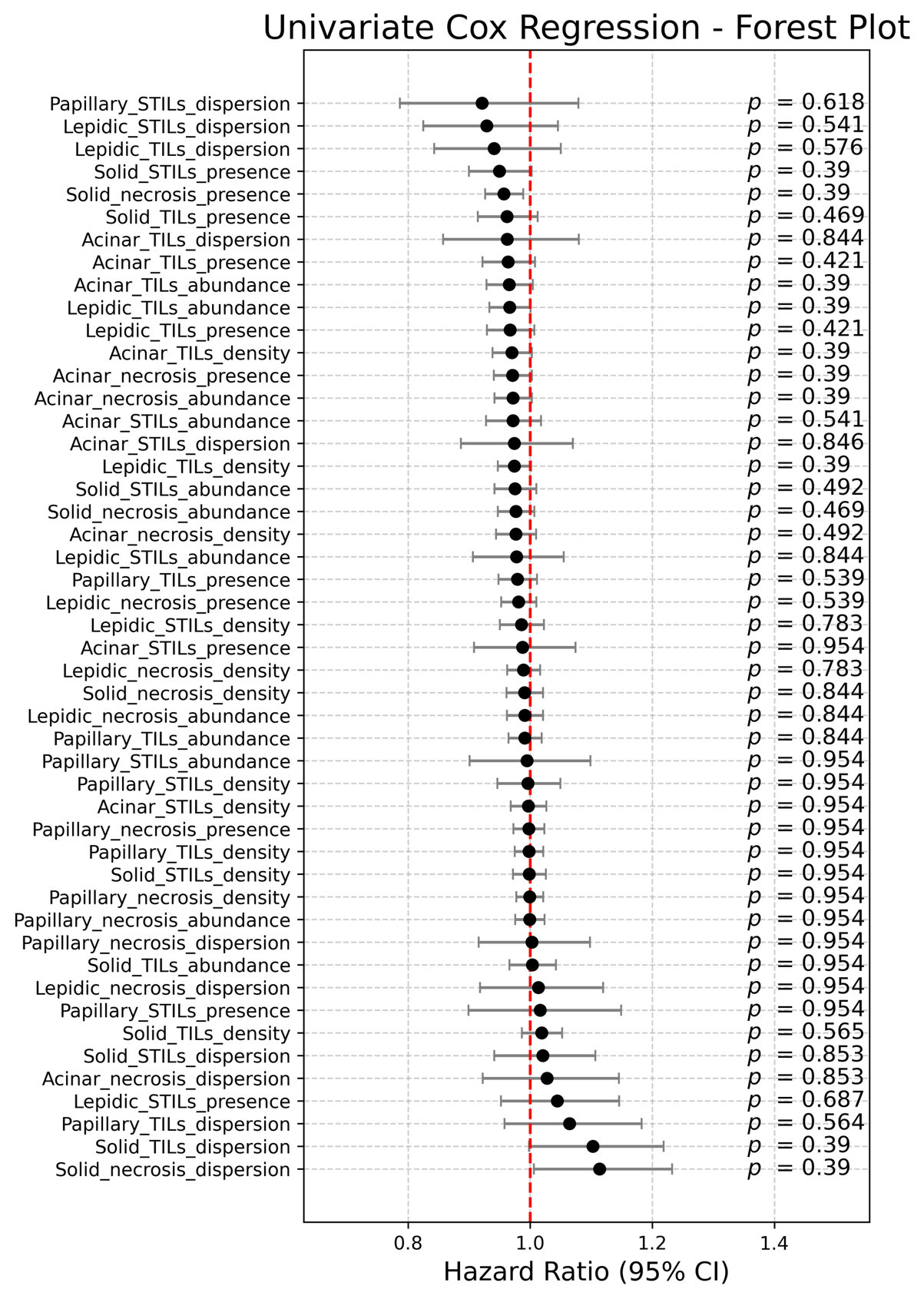


**Figure S4**. **Forest plot for univariate analysis using the Cox proportional hazards model and overall survival as the endpoint for the proposed features.** p-values adjusted for multiple comparisons using the Benjamini–Hochberg correction.


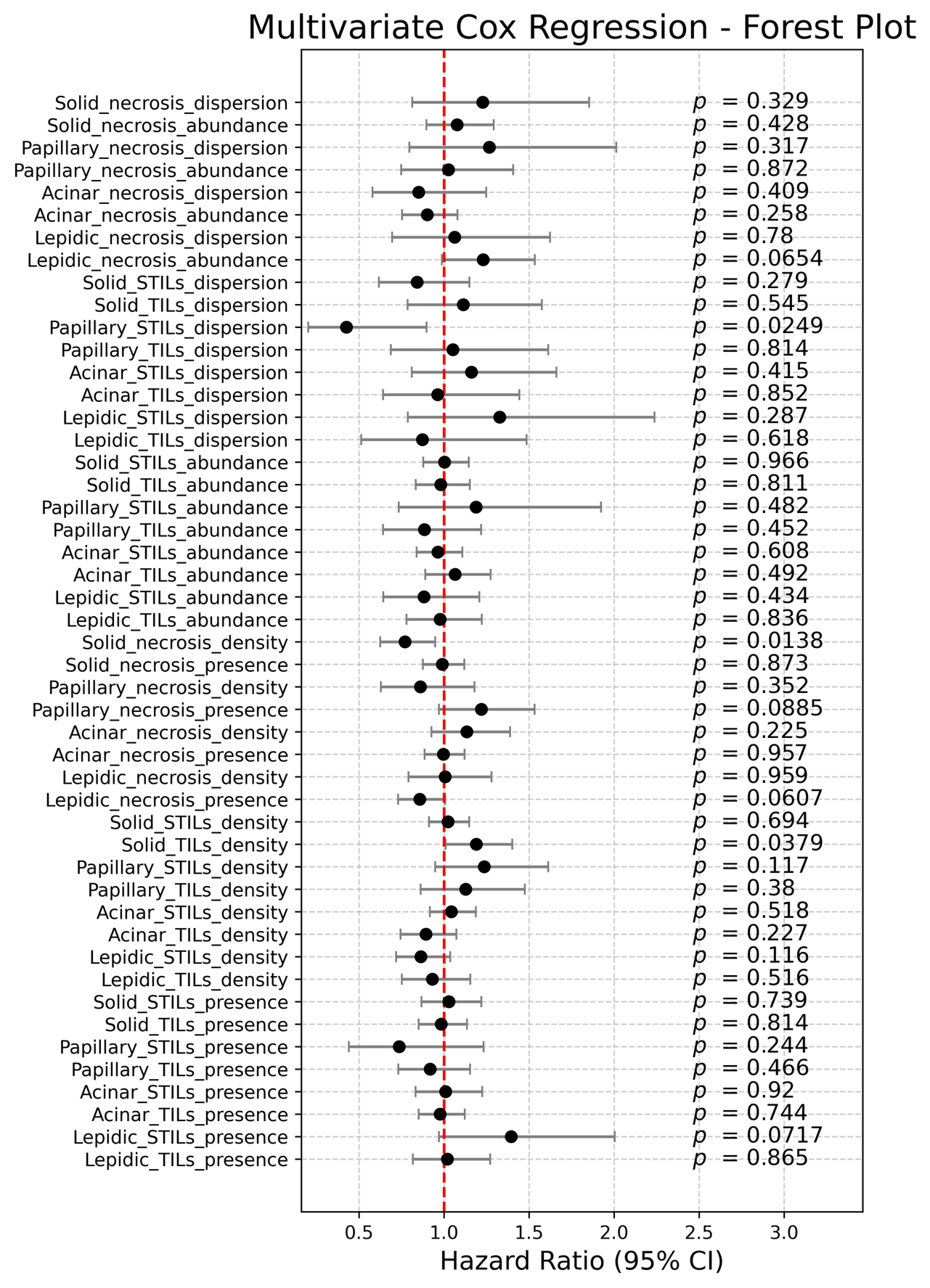


**Figure S5**. **Forest plots for multivariate analysis using the Cox proportional hazards model and overall survival as the endpoint for the proposed features.**


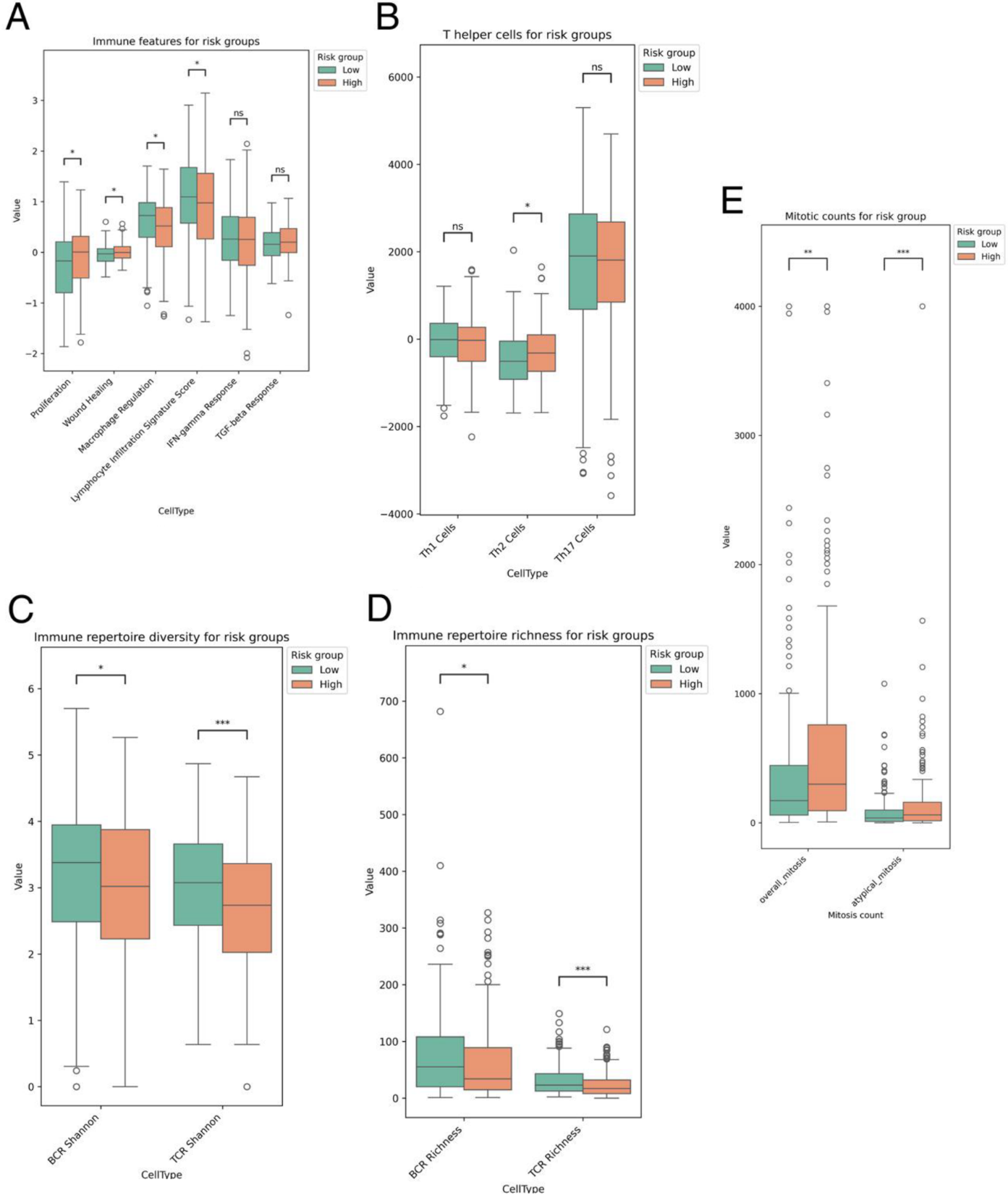


**Figure S6**. **Boxplots showing the distribution of different tumour immune microenvironment features across predicted the low-risk and high-risk groups.** Statistical significance between groups was assessed using Mann–Whitney U-tests, with p-values adjusted for multiple comparisons using the Benjamini–Hochberg correction. p-values are denoted as asterisk (*p-value < 0.05, **p-value < 0.01, ***p-value < 0.001, ns: p-value > 0.05).


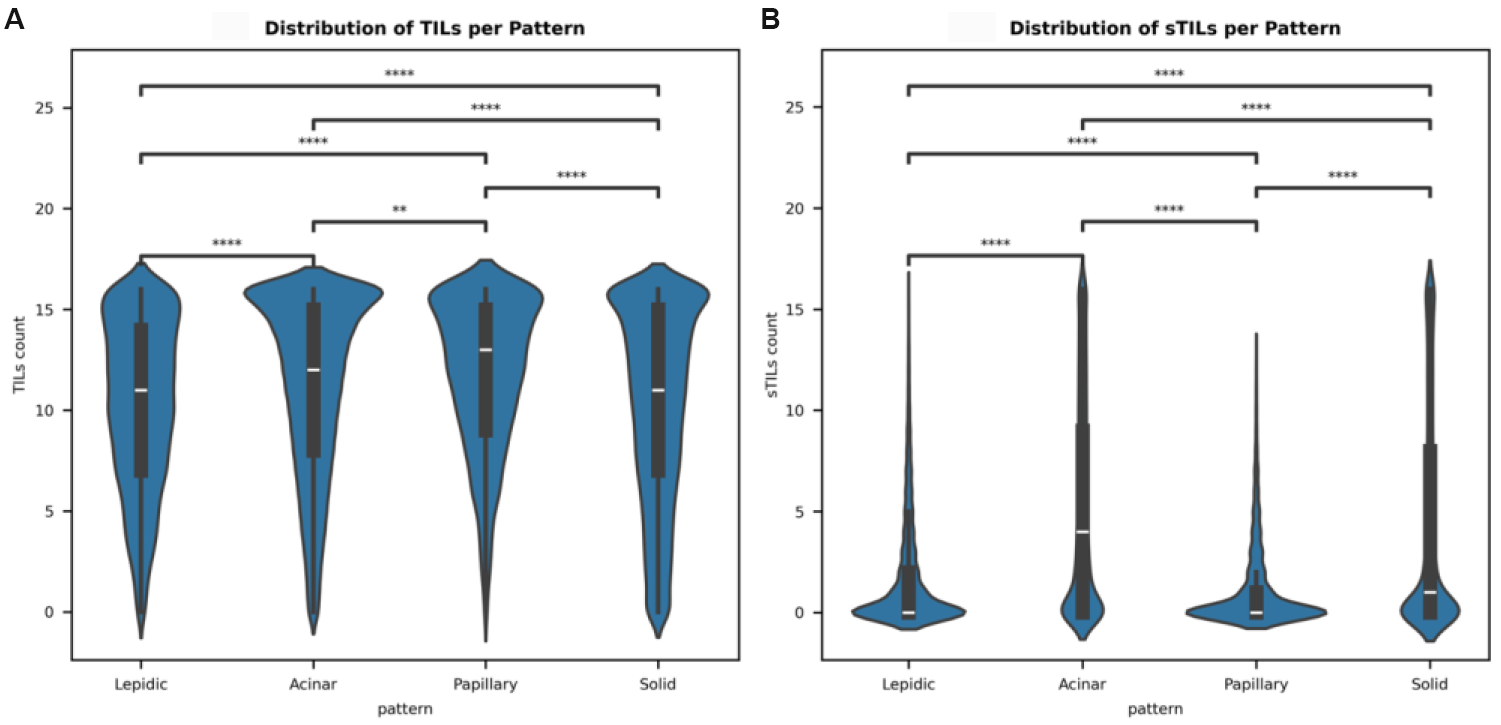


**Figure S7**. **Violin plots showing the distribution of TILs and sTILs counts in the different growth patterns in the entire cohort.** *0.01 < p ≤ 0.05, **0.001 < p ≤ 0.01, ***0.0001 < p ≤ 0.001, ****p ≤ 0.0001.


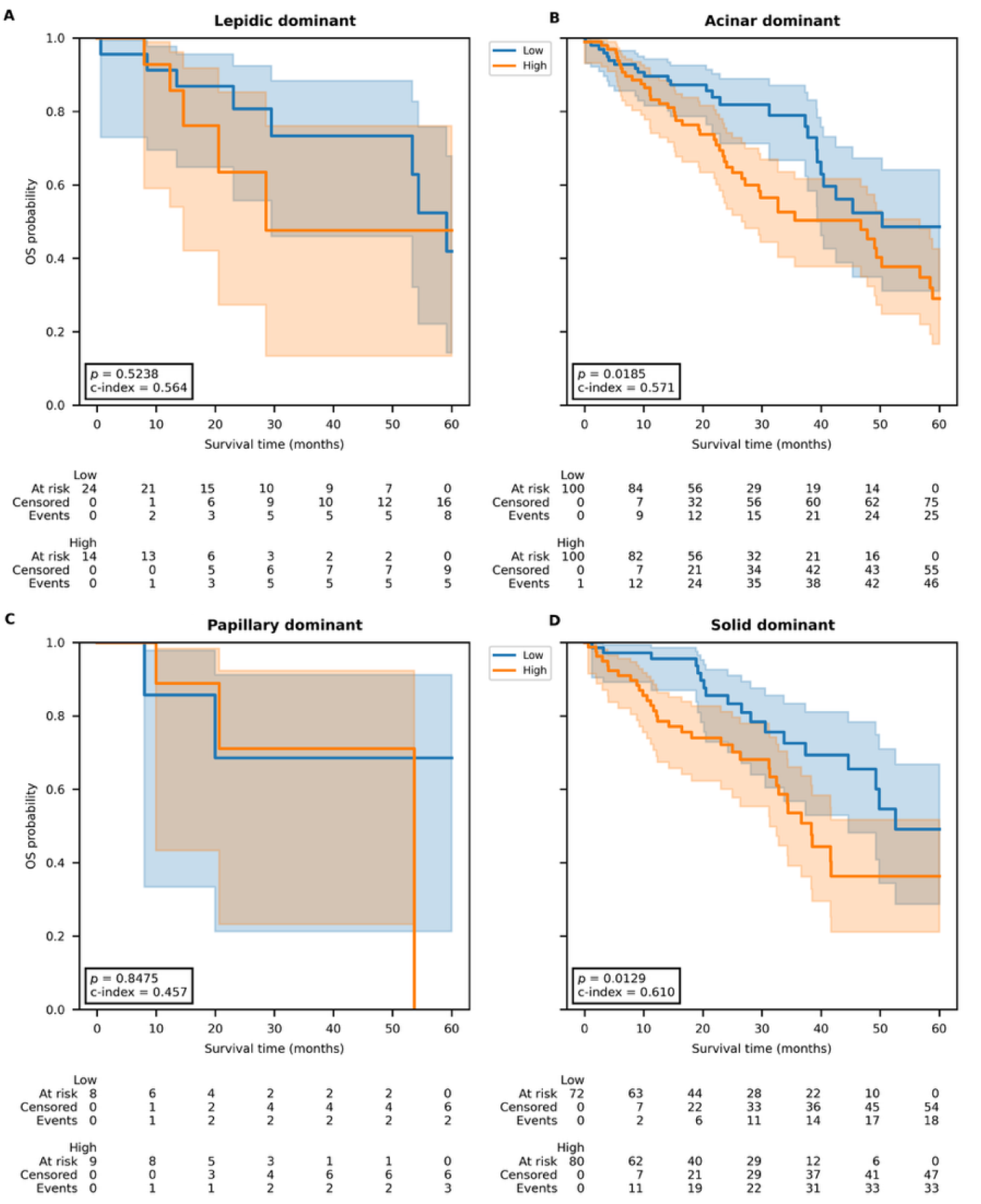


**Figure S8**. **Kaplan–Meier curves for GPS-TILs marker stratified by the dominant pattern.**


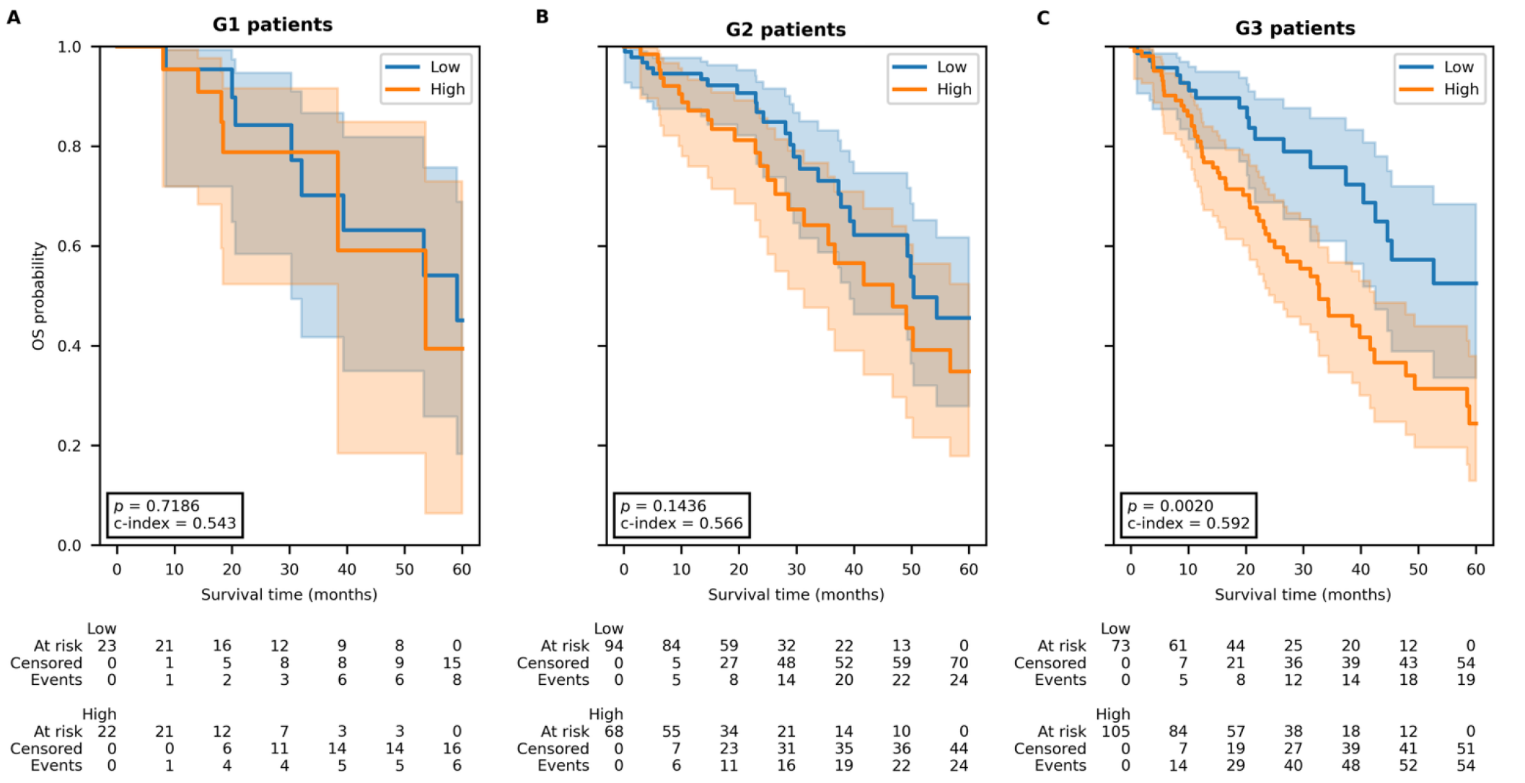


**Figure S9.** **Kaplan–Meier curves for GPS-TILs marker stratified by grade.**


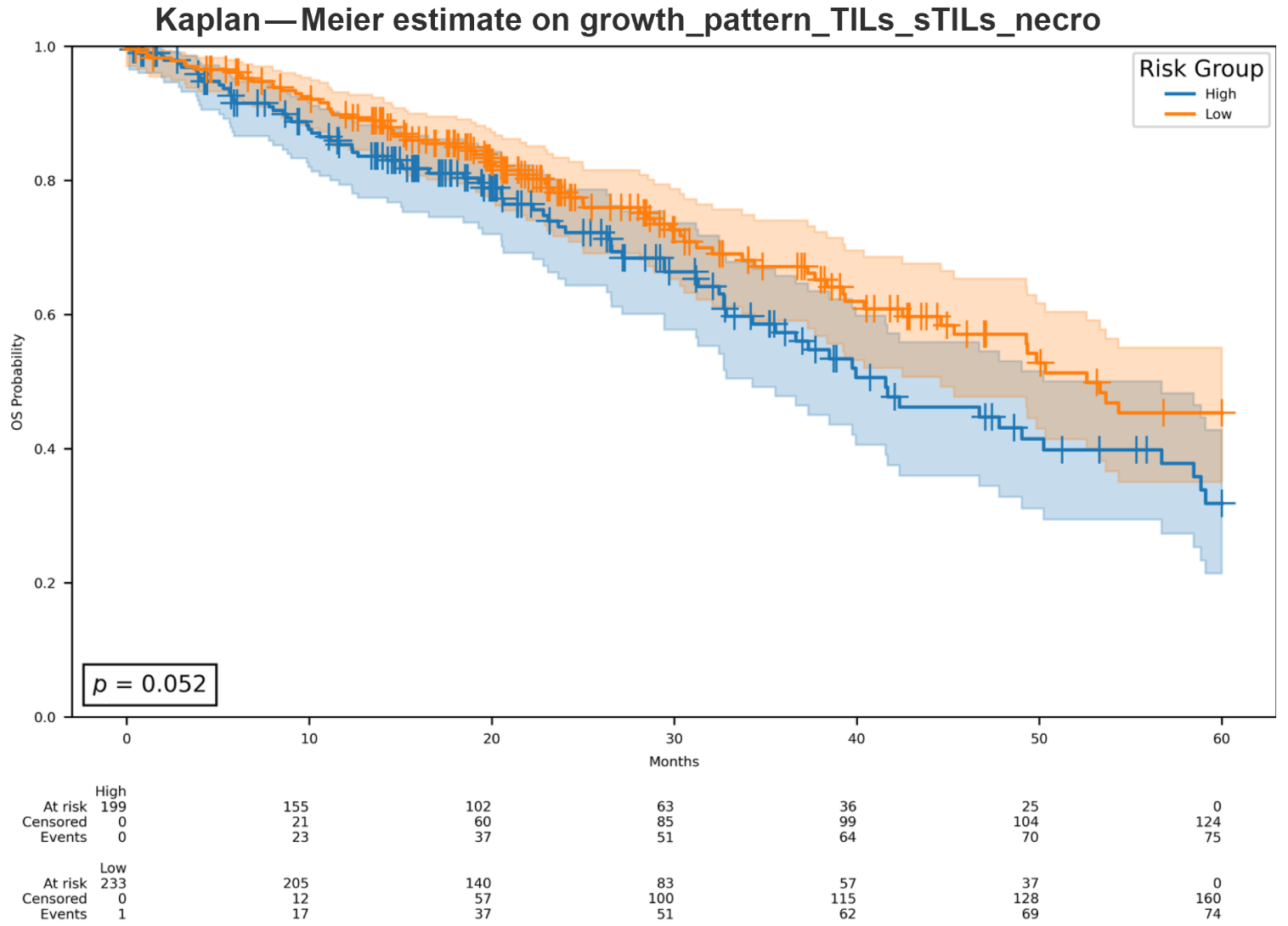


**Figure S10.** **Kaplan–Meier (KM) survival curves for cross-validation results using: TILs, sTILs, and necrotic area in different growth patterns (including micropapillary), C-index = 0.57.**


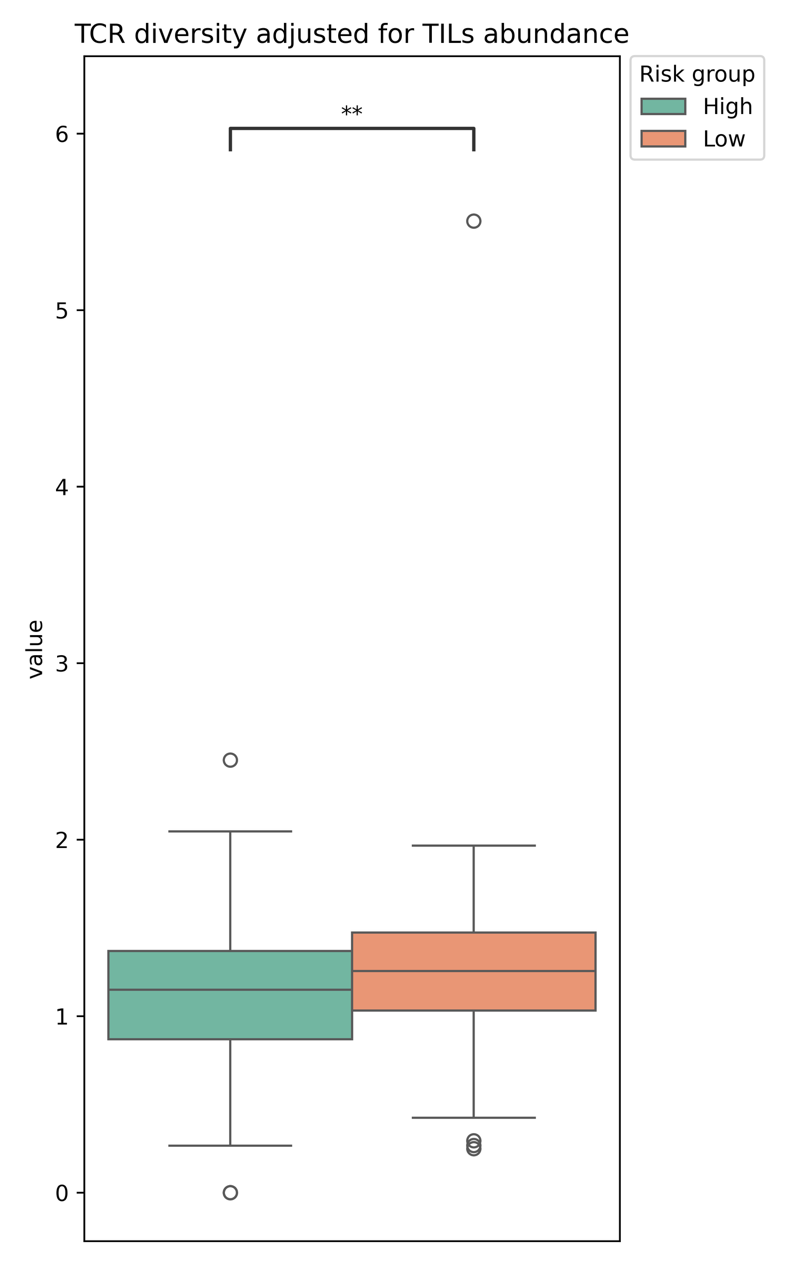


**Figure S11.** **Boxplot showing the distribution of the TCR Shannon normalized by log-transformed TILs abundance for predicted low-risk and high-risk groups.** Statistical significance between groups was assessed using Mann–Whitney U-tests, with p-values adjusted for multiple comparisons using the Benjamini–Hochberg correction. p-values are denoted as asterisk (*p < 0.05, **p < 0.01, ***p < 0.001, ns: p > 0.05)

**Table S1.** Description of the features used to derive the digital biomarker.

| **Feature name** | **Description** | **Calculation** |
| --- | --- | --- |
| TILs presence | Number of inflammatory cells in the least immune active areas of the tumour | ${= min}_{i=1,\ldots,N}{TILs}_{i}$ |
| TILs density | Density of inflammatory cells in the immune active area areas of the in tumour | $= {max}_{i=1,\ldots,N}{TILs}_{i}$ |
| TILs abundance | Overall density of inflammatory cells in the tumour area | $\mu_{TILs}= \frac{1}{N} \sum_{i=1}^{N} {TILs}_{i}$ |
| TILs dispersion | Dispersion of inflammatory cells in the tumour area | $\sigma_{TILs}= \sqrt{\frac{1}{N} \sum_{i=1}^{N} {({TILs}_{i}- \mu_{TILs})}^{2}}$ |
| sTILs presence | Number of inflammatory cells in dense stroma area within or around the tumour with the least immune activity | ${= min}_{i=1,\ldots,N}{sTILs}_{i}$ |
| sTILs density | Density of inflammatory cells in dense stroma area within or around the tumour | $= {max}_{i=1,\ldots,N}{sTILs}_{i}$ |
| sTILs abundance | Overall density of inflammatory cells in dense stroma area within or around the tumour | $\mu_{STILs}= \frac{1}{N} \sum_{i=1}^{N} {sTILs}_{i}$ |
| sTILs dispersion | Dispersion of inflammatory cells in dense stroma area within or around the tumour | $\sigma_{STILs}= \sqrt{\frac{1}{N} \sum_{i=1}^{N} {({sTILs}_{i}- \mu_{STILs})}^{2}}$ |
| Necrosis presence | The smallest number of localized necrotic cells in or adjacent to the tumour. | ${= min}_{i=1,\ldots,N}{Necro}_{i}$ |
| Necrosis density | The largest number of localized necrotic cells in or adjacent to the tumour. | $= {max}_{i=1,\ldots,N}{Necro}_{i}$ |
| Necrosis abundance | Overall density of necrotic cells. | $\mu_{Necro}= \frac{1}{N} \sum_{i=1}^{N} {Necro}_{i}$ |
| Necrosis dispersion | Dispersion of necrotic cells. | $\sigma_{Necro}= \sqrt{\frac{1}{N} \sum_{i=1}^{N} {({Necro}_{i}- \mu_{Necro})}^{2}}$ |

TILsi is the number of TILs-labelled tiles in patch i. sTILsi is the number of sTILs-labelled tiles in patch i. Necroi is the number necrotic tiles in patch i. N is the number of patches in a WSI.

**Table S2.** Risk score association with key clinicopathological features

| **Feature** | ***p*-value** | **Correlation Strength** |
| --- | --- | --- |
| Diagnosis age | 0.18740 | −0.0651 |
| Packs smoked/year | 0.75340 | −0.0180 |
| Number of years smoked | 0.51270 | 0.0504 |
| Lepidic percentage | 0.02510 | −0.1077 |
| Papillary percentage | 0.15720 | −0.0681 |
| Acinar percentage | 0.58700 | −0.0262 |
| Solid percentage | 0.02120 | 0.1108 |
| AJCC Pathologic T-Stage | 0.17080 | - |
| AJCC Pathologic N-Stage | < 0.00001 | - |
| AJCC Pathologic M-Stage | 0.72510 | - |
| AJCC Pathologic Stage | 0.00220 | - |
| Ethnicity | 0.86770 | - |
| Sex | 0.02040 | 0.0204 |

**Table S3.** Average and standard deviation of per class accuracy and F1 for CellOMaps on TCGA-LUAD using patient-level cross-validation.

| **Measure** | **Lepidic** | **Acinar** | **Papillary** | **Micropapillary** | **Solid** |
| --- | --- | --- | --- | --- | --- |
| **Accuracy** | 0.77 ± 0.09 | 0.74 ± 0.11 | 0.45 ± 0.20 | 0.51 ± 0.13 | 0.87 ± 0.13 |
| **F1** | 0.87 ± 0.05 | 0.84  ± 0.08 | 0.59 ± 0.21 | 0.67 ± 0.11 | 0.93 ± 0.08 |
